# “Pay them if it works”: Discrete choice experiments on the acceptability of financial incentives to change health related behaviour

**DOI:** 10.1016/j.socscimed.2012.09.033

**Published:** 2012-12

**Authors:** Marianne Promberger, Paul Dolan, Theresa M. Marteau

**Affiliations:** a Health Psychology Section, Department of Psychology, King's College London, UK; b Department of Social Policy, London School of Economics, UK

**Keywords:** UK, Health incentives, Acceptability, Public policy

## Abstract

The use of financial incentives to change health-related behaviour is often opposed by members of the public. We investigated whether the acceptability of incentives is influenced by their effectiveness, the form the incentive takes, and the particular behaviour targeted. We conducted discrete choice experiments, in 2010 with two samples (*n* = 81 and *n* = 101) from a self-selected online panel, and in 2011 with an offline general population sample (*n* = 450) of UK participants to assess the acceptability of incentive-based treatments for smoking cessation and weight loss. We focused on the extent to which this varied with the type of incentive (cash, vouchers for luxury items, or vouchers for healthy groceries) and its effectiveness (ranging from 5% to 40% compared to a standard treatment with effectiveness fixed at 10%). The acceptability of financial incentives increased with effectiveness. Even a small increase in effectiveness from 10% to 11% increased the proportion favouring incentives from 46% to 55%. Grocery vouchers were more acceptable than cash or vouchers for luxury items (about a 20% difference), and incentives were more acceptable for weight loss than for smoking cessation (60% vs. 40%). The acceptability of financial incentives to change behaviour is not necessarily negative but rather is contingent on their effectiveness, the type of incentive and the target behaviour.

## Highlights

► Effectiveness of incentive schemes for healthier behaviour increased their acceptability, in studies conducted with UK participants. ► Even a small increase of effectiveness, from 10% to 11%, increased acceptability. ► Grocery vouchers were better accepted than cash or luxury item vouchers. ► Incentives were better accepted for weight loss than for smoking cessation.

## Introduction

Smoking, poor diet, and physical inactivity contribute to a range of chronic diseases and explain much of the variation in premature mortality (Beaglehole et al., 2011). Health care providers seek effective ways to change these behaviours. There is increasing interest in programmes that pay individuals contingent upon changing their health-related behaviour (Marteau, Ashcroft, & Oliver, 2009). There is evidence of effectiveness in smoking cessation in pregnancy (Bauld & Coleman, 2009; Lumley et al., 2009) and drug treatment programmes (Lussier, Heil, Mongeon, Badger, & Higgins, 2006) but there is currently less evidence regarding their effectiveness in weight loss programmes (Volpp et al., 2008) and programmes for general smokers (Cahill & Perera, 2008; Volpp et al., 2006) with one exception, which used large incentives (Volpp et al., 2009).

Such programmes have provoked negative reactions from the public, especially when they involve cash payments (Long, Helweg-Larsen, & Volpp, 2008; Parke, Ashcroft, Brown, Marteau, & Seale, 2011; Priebe et al., 2010). Given equally effective alternative treatments, incentive-based ones are less acceptable to US and UK participants (Promberger, Brown, Ashcroft, & Marteau, 2011). In a qualitative study assessing attitudes towards using financial incentives for medication adherence, however, many participants stated that effectiveness was the most important factor in determining the acceptability of offering financial incentives (Priebe et al., 2010, p. 466), and in a recent poll, acceptability of plain cigarette packaging increased with increasing effectiveness (ASH, 2011).

Against this background, the current studies aim to investigate the extent to which people are willing to trade off dislike of incentive programmes against an explicit gain in treatment effectiveness. We focus on smoking cessation and weight loss, two of the health contexts for which there is much interest in using financial incentives to improve population health (Cahill & Perera, 2008; Paul-Ebhohimhen & Avenell, 2008).

## Methods

We conducted three studies, the third built on the first two. Studies 1 and 2 were conducted online in 2010 and Study 3 was conducted in 2011 by a research agency in high streets in five UK cities. The research was approved by the King's College London PNM ethics committee (online studies; PNM/09/10-89) and covered by the professional code of conduct of the Market Research Society (for the research agency). In all three studies, we used discrete choice experiments (DCEs) to assess how readily accepted were incentive-based treatments of different types and levels of effectiveness relative to standard treatments of a fixed level of effectiveness. DCEs ask participants to select among several options, across a range of choice sets to reveal strength of preferences and how these are affected by the factors varied in the DCE.

### Design

Studies 1 and 2 assessed acceptability of incentive-based treatments for smoking cessation and weight loss, respectively. Table 1 shows the factors and levels. Each combination of incentive type and incentive effectiveness was presented against a constant comparator of “standard medication” at 10% effectiveness, resulting in a three (incentive effectiveness) × three (incentive type) within-subject design. Ten percent effectiveness was chosen because it is a broadly plausible level of effectiveness for both conditions (Padwal, Rucker, Li, Curioni, & Lau, 2003; Shaw, Gennat, O'Rourke, & Del Mar, 2006; Stead, Perera, Bullen, Mant, & Lancaster, 2008).

Study 3 built on the online studies to replicate and extend the findings in an offline sample. We included both treatment contexts in one study to enable direct comparison. We also included a factor level for which the incentive is less effective than the constant comparator (5% vs. 10%), to compare with trade-offs in the other direction, and one for which the effectiveness increase is very small (11% vs. 10%), to assess sensitivity to small effectiveness increases. To keep the number of choice pairs manageable, we dropped the incentive type of luxury item vouchers, as its effect was no different to cash in Studies 1 and 2. This gave a two (treatment context) × five (incentive effectiveness) × two (incentive type) within-subject design. We changed the constant comparator wording to “standard treatment” for weight loss, to avoid any potential influence of lack of familiarity of medication-based treatments in this context.

### Material

Table 2 shows an example of a choice pair. When the treatment context was weight loss, incentives were for “meeting weight loss targets” and standard medication or treatment “for weight loss”. £50 per month was the same for all choice pairs, and is an amount typical for such incentive programmes (e.g., Ballard & Radley, 2009: £12.50 per week; Marteau, Thorne, Aveyard, Hirst, & Sokal, submitted for publication: £752 over 58 weeks). We made the standard treatment of equivalent cost.

For each treatment context, we added one choice pair as a dominance check comparing standard treatment at 10% effectiveness with standard treatment at 20% effectiveness. This check and intransitive choices (rejecting the incentive, but accepting it at lower effectiveness) were measures of inconsistency.

Studies 1 and 2 were conducted online. In each part, the 10 choice pairs were presented one at a time; the order was randomized for each participant, as was the position of the constant comparator (left vs. right) in each pair. This was followed by additional questions about the topic and about gender, age, and education.

For Study 3, the material was in a booklet, starting with the choice tasks, followed by questions about the topic and about gender, age, education, and postcode. We asked whether participants were overweight (never – formerly – slightly – very) and whether they smoked (never – have quit – not daily – daily). The 22 choice pairs were grouped by treatment context; order of the contexts was randomized. Within each context, the eleven choice pairs were presented in one of four different randomly generated orders. The position of the constant comparator alternated (left vs. right).

All study material is available as Supplementary material online.

### Participants and procedure

For Studies 1 and 2, participants were recruited through an online panel (http://participate-in-research.org.uk). Only panel members registered as UK residents aged over 18 years were contacted; for Study 2 nobody was contacted who had completed Study 1. Participants were sent an email with a link to the study and paid £3 (US$ 4.8) for completion through the panel. Panel members self-select by signing up to the website to “participate in online academic research and get paid a small amount of money.” The resulting sample is a convenience sample comprised of members of the general public with an internet connection. About 400 participants were invited to Study 1 and were informed that recruitment would close at 80 participants. About 300 participants were invited to Study 2 and informed recruitment would close at 100 participants. Each study closed after about 48 h.

For Study 3, participants were recruited through a research agency (Wyman Dillon, http://wymandillon.co.uk) in five cities across the UK (Birmingham, Bristol, London, Manchester, Northampton), aiming for similar numbers of men and women and participants of different education levels. Participants were approached in a high street and asked whether they would take part in a survey about health care treatments. They completed the questionnaire on their own at desks in a venue rented for the purpose and were paid £4 (US$6.4) for their participation.

### Data analysis

We used mixed effects logistic regression models with participant random effects to analyze choices (Bates, Maechler, & Bolker, 2011; R Development Core Team, 2011). Random effects account for the non-independence of data from the same participants (Probit models gave qualitatively identical results).

Choice of the standard medication or treatment was coded as 0 and choice of the incentive as 1. Incentive type and incentive effectiveness were independent variables. For Study 3, treatment context was an additional independent variable, as was the interaction between treatment context and incentive type. The models estimate relative preference of the incentive treatment at different factor levels over standard treatment at 10% effectiveness. Details of the analysis and models are available from the authors.

In addition, to judge absolute acceptance of the incentive relative to standard treatment at the same level of effectiveness, we used Chi–Square tests to test the null hypothesis that 50% choose the incentive.

We excluded participants from the analyses who may not have understood the task or not taken it seriously, based on the dominance check, on intransitive choices and on response times in the online studies (see supplementary material).

We included non-traders (participants always choosing standard treatment regardless of incentive effectiveness), given that some people are strongly opposed to health incentives (Parke et al., 2011; Promberger et al., 2011). Our aim was to establish whether and how people trade off their dislike for incentives against effectiveness, therefore we included participants who never accepted incentive treatments, even when they were much more effective.

## Results

### Participants

Table 3 shows the characteristics of participants in all studies.

### Study 1 and Study 2

**Fig. 1:**
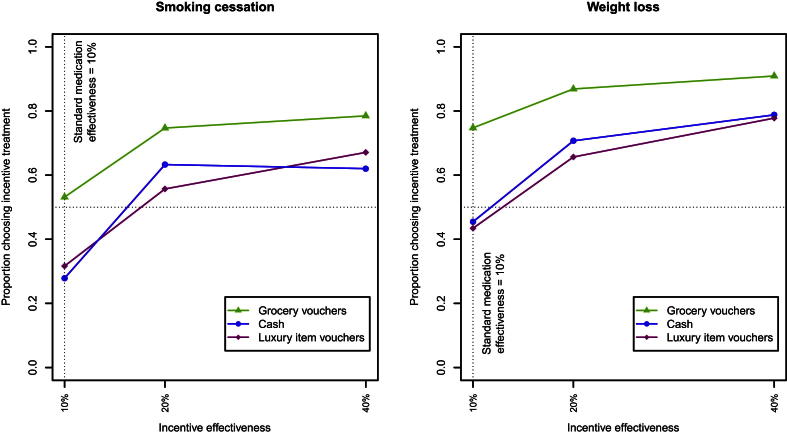
Proportion of participants choosing the incentive treatment over standard medication at 10% effectiveness in Study 1 (smoking cessation) and Study 2 (weight loss).

Fig. 1 shows the proportions of participants choosing the incentive-based treatments in each condition in Study 1 and Study 2.

Table 4 shows the model coefficients representing the influence of the incentive treatment characteristics on whether it is chosen over 10% effective standard medication. The model uses treatment contrasts for the categorical variables, comparing effectiveness levels 20 and 40% each with the reference level 10%, and luxury item and grocery vouchers each with cash. A positive coefficient corresponds to that factor level strengthening preference for the incentive. Age, gender and education had no effect when included and are not included in these models.

For both smoking cessation and weight loss, increasing incentive effectiveness to 20% and 40% each significantly increases its acceptability compared to incentive-based treatment at 10% effectiveness. As Fig. 1 shows, this effect is non-linear: increasing effectiveness to 20% has a proportionately larger effect than increasing it to 40%.

For smoking cessation, cash (*Χ*^2^ = 15.50, *p* < 0.001) and luxury voucher incentives (*Χ*^2^ = 10.65, *p* < 0.01) are less well accepted than equally effective standard medication, but vouchers for healthy groceries are accepted equally well as standard medication. For weight loss, there is no evidence for lower acceptability of cash or luxury item vouchers, and grocery vouchers are better accepted than equally effective standard treatment (*Χ*^2^ = 20.60, *p* < 0.001).

For smoking cessation and weight loss, 11 (14%) and 4 (4%) participants always rejected the incentive-based treatment, respectively (“non-traders”).

A further 21 (27%) consistently refused at least one incentive type for smoking cessation, most of them cash (14); 21 (21%) did so for weight loss, most of them luxury item vouchers (14).

### Study 3

**Fig. 2:**
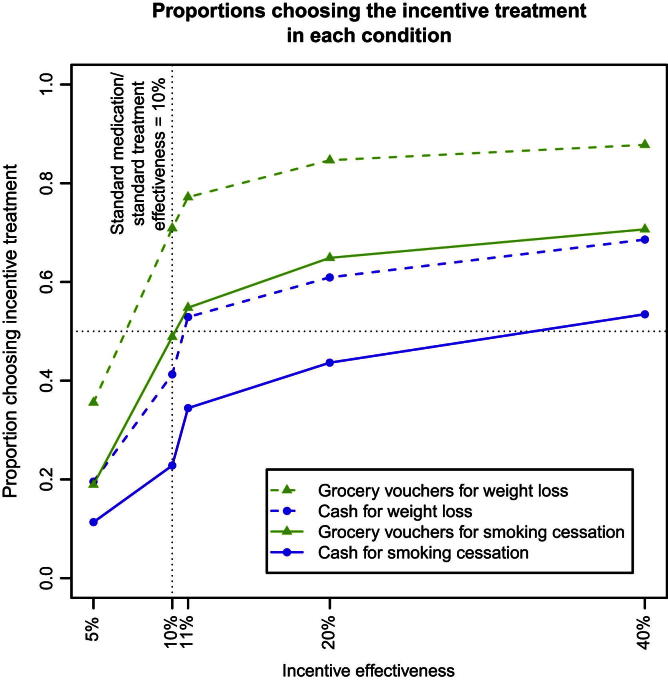
Proportion of participants choosing the incentive in each condition over standard medication or standard treatment at 10% effectiveness in Study 3.

Fig. 2 shows the proportion of participants choosing the incentive treatment over standard treatment at 10% effectiveness in each condition in Study 3; model coefficients are in Table 5, with reference levels “10% effectiveness”, “cash” and “smoking cessation” for the categorical variables. Age, gender and education had no effect and are not included in the model.

As in Studies 1 and 2, increasing the effectiveness of an incentive increases its acceptability and this effect is again non-linear. Notably, in aggregate, participants are sensitive even to the small increase in effectiveness from 10% to 11%. Not surprisingly, lowering the effectiveness of the incentive to 5% reduces its acceptability.

Incentives are more acceptable for weight loss. Grocery vouchers are more acceptable than cash in both contexts, and they increase acceptability in the context of weight loss more than they do for smoking cessation (interaction term). At equal effectiveness, cash incentives are less acceptable than standard treatment for smoking cessation (*Χ*^2^ = 132.10, *p* < 0.001) and for weight loss (*Χ*^2^ = 13.58, *p* < 0.001). For weight loss, grocery vouchers are more acceptable than standard treatment (*Χ*^2^ = 78.54, *p* < 0.001).

Separate models including participants' overweight and smoking status and their interaction with incentive type show that both the very overweight and daily smokers are more in favour of incentive-based treatments than those who were never overweight or never smoked (see Table 6).

26 participants (6%) always rejected the incentive-based treatment, regardless of effectiveness, type, or context (“non-traders”). A further 184 (41%) consistently refused at least one combination of incentive type and context at any effectiveness, most of them (154) cash for smoking cessation.

## Discussion

The acceptability of incentive-based treatments increases with effectiveness: most participants were willing to trade off their dislike of incentive treatments against effectiveness. Acceptability also varied with incentive type, with grocery vouchers seen as more acceptable than cash or luxury items. Cash incentives for smoking cessation were less acceptable than standard treatment. All incentives were more acceptable for weight loss than for smoking cessation. Grocery vouchers were particularly well accepted for weight loss.

Lower acceptability of cash incentives for smoking cessation than standard treatment was also found in a previous study among UK and US participants (Promberger et al., 2011) and mirrors media portrayals (Parke et al., 2011). It is also reflected by non-smokers rejecting an effective programme for smokers employed in the same organization (Volpp, Asch, Galvin, & Loewenstein, 2011). The higher acceptability of incentives for weight loss may reflect different levels of “moralization” of the two behaviours, a process conferring moral significance to health-related behaviours (Rozin, 1999). Such moralizing could explain higher acceptance of grocery vouchers than cash, with the former seen less as a “reward” for morally reprehensible behaviour.

This is supported by grocery vouchers being particularly well accepted for weight loss (Study 3). As we described them, vouchers for “healthy groceries” may have been seen as instrumental for weight loss, and seen as a treatment rather than a reward. The lower acceptability of smoking cessation incentives by those who never smoked than by those who smoked daily, and lower acceptability of weight loss incentives by those who were never overweight than the very overweight, might also reflect moralizing among those not afflicted. Alternatively, it could reflect self-interest of smokers and overweight participants in being offered such rewards. Previously, Long et al. (2008) had also found smokers and obese patients to be more likely to endorse incentive programmes than non-smokers and non-obese respondents. Two components underlie the non-linear effect of effectiveness on acceptability: first, some participants are very sensitive to effectiveness, and even an increase of one percentage point overrides their dislike of financial incentives; second, some participants are strongly opposed to incentives and are unwilling to accept them even at 40% effectiveness. Such a “vocal minority” might impede implementation of incentives (Volpp et al., 2011).

10% standard treatment effectiveness may have lowered participants' perceptions of standard treatment effectiveness compared to their perceptions had they not been given explicit information. In this case, providing only information on incentive effectiveness would likely lower acceptance of incentive treatments. On the other hand, acceptance might be higher if the standard vs. incentive effectiveness difference were presented in relative rather than absolute terms (“100% increase in effectiveness”).

To our knowledge, this is the first study examining how acceptability of incentives varies with their stated effectiveness, and the first study comparing the acceptability of different incentive types. We specified simplified monthly costs of £50 for both incentive and standard treatment. As this does not capture the full costs of either intervention, we relied on participants ignoring other costs, or assuming them to be similar across treatment types. Specifying costs for both treatments increases the validity of our findings: the cost of standard treatment may otherwise be neglected compared to that of financial incentives. The choice between alternatives with the same cost and clearly stated effectiveness may result in more favourable views of incentive programmes than other methods of preference elicitation such as direct ratings of incentive programmes, more commonly used by media and opinion polls.

Participants may have interpreted grocery vouchers for weight loss to have higher than stated effectiveness, and future studies should attempt to check for this by assessing perceived effectiveness. Our study was conducted with UK participants. Promberger et al. (2011) have previously found very similar attitudes to incentive treatments in UK and US participants. The study would merit replication in a US sample to confirm whether the results would be applicable to US health policy decisions.

If public policy aims to maximize health benefits, this means funding the most cost-effective treatment for any given health condition. Dislike of incentives may reflect public preferences for non-health related concerns about fairness and equity (e.g., Dolan, Shaw, Tsuchiya, & Williams, 2005). Such preferences are not always well-reflected and consistent (Ubel, Baron, & Asch, 2001), and we do not address the question of how they *should* be traded off against effectiveness. However, our studies show that in aggregate, participants *do* make these trade-offs. If incentive-based treatments are found to be effective, this should be clearly communicated to improve the acceptability of effective treatments. As shown, even small increases in effectiveness may considerably boost acceptability. Our studies also show vouchers for healthy groceries are better accepted than cash; making them preferable if equally effective. This finding might extend to other incentives with health promoting characteristics; future research should establish which incentive characteristics have this effect.

Future research about attitudes towards health incentives, and indeed about attitudes to other novel health interventions such as plain packaging for cigarettes, should take into account people's willingness to trade off initial opposition to them against increased effectiveness. When assessing acceptability of incentive-based programmes, the cost of standard medication should be stated to make treatments comparable. A minority of participants are not willing to accept incentive treatments even if they are four times as effective as standard treatment. Future research should improve the understanding of their motivations and implications for policy: concerns about consequences such as motivation “crowding out” call for establishing evidence about these consequences, while reluctance to reward “immoral” health behaviours of the day requires judgement how to weigh such sentiments against tangible consequences.

Notwithstanding these issues, these studies have shown a clear preference, in our UK samples, for using financial incentives to change health-related behaviours when those incentives are shown to be more effective than other behaviour change interventions and when they do not involve cash.

## Figures and Tables

**Table 1:** Discrete choice experiment factors and levels used in Studies 1, 2 and 3.

Factor	Levels in Study 1 and Study 2	Levels in Study 3
Incentive effectiveness	–	5%,
	10%,	10%,
	–	11%,
	20%,	20%,
	40%	40%
Incentive type	Cash	Cash
	Vouchers for luxury items	–
	Vouchers for healthy groceries	Vouchers for healthy groceries

Constant comparator	Standard medication [treatment] at 10% effectiveness	

**Table 2:** Example of a choice pair for Study 1.

Treatment A	Treatment B
The patient receives	The patient receives
vouchers for healthy groceries	standard medication
worth £50 per month for not smoking.	worth £50 per month for stopping smoking.
This intervention is proven to help	This treatment is proven to help
20 out of 100 treated.	10 out of 100 treated.

Which treatment should be funded?	
[ ] Treatment A	
[ ] Treatment B	

**Table 3:** Demographic characteristics of study participants.

		Study 1	Study 2		Study 3
Online?		Online	Online		Offline
Original sample size		*n* = 81	*n* = 101		*n* = 517
*n* after exclusions based on inconsistency checks (representing x% of original sample)		79 (96%)	99 (98%)		450 (87%)
Mean age in years (SD)		46.82 (14.89)	44.07 (14.54)		43.77 (17.53)
Women (*n*(%))		47 (58%)	58 (59%)		217 (48%)
Education (*n*(%)):					
	No qualifications	5 (6%)	5 (5%)		87 (19%)
	GCSE	18 (23%)	12 (12%)		81 (18%)
	A-levels	13 (16%)	26 (26%)		60 (13%)
	Higher	9 (11%)	19 (19%)		61 (14%)
	Degree	33 (42%)	34 (34%)		123 (27%)
	Other	1 (1%)	3 (3%)		21 (5%)
Smoking status (*n* (%))					
				Currently smoke daily	90 (20%)
	Currently smoke	21 (27%)		Currently smoke, but not daily	19 (4%)
	Have quit	23 (29%)		Have quit	118 (16%)
	Never	30 (38%)		Never	216 (48%)
Overweight status (*n* (%))					
				Very overweight	44 (10%)
	Overweight	47 (47%)		Slightly overweight	163 (36%)
	Formerly overweight	20 (20%)		Formerly overweight	46 (10%)
	Never	32 (32%)		Never	188 (42%)

**Table 4:** Model coefficients and confidence intervals for Study 1 and Study 2.

Factor/Factor level	Coefficients [95% confidence interval]	
	Study 1 (smoking cessation)	Study 2 (weight loss)
Intercept: Effectiveness: 10% Type: cash	−1.68*** [−2.53, −0.83]	−0.16 [−0.81, 0.49]
Effectiveness: 20%	2.36*** [1.77, 2.94]	1.73*** [1.24, 2.22]
Effectiveness: 40%	2.81*** [2.20, 3.42]	2.61*** [2.06, 3.16]
Type: grocery vouchers	1.70*** [1.12, 2.28]	2.00*** [1.44, 2.55]
Type: luxury item vouchers	0.04 [−0.49, 0.56]	−0.23 [−0.69, 0.23]
Model based on *n* observations (*n* participants)	711 (79)	891 (99)

*P*-values based on Wald tests. *** < 0.001.

**Table 5:** Model coefficients and confidence intervals for Study 3.

Factor/Factor level	Coefficients [95% confidence interval]
Intercept: Effectiveness: 10% Type: cash Context: smoking cessation	−1.54*** [−1.76, −1.32]
Effectiveness: 5%	−1.75*** [−1.93, −1.56]
Effectiveness: 11%	0.56*** [0.39,0.72]
Effectiveness: 20%	1.14*** [0.97,1.31]
Effectiveness: 40%	1.61*** [1.43, 1.79]
Type: grocery vouchers	1.24*** [1.09, 1.40]
Context: weight loss	1.04*** [0.89, 1.20]
Interaction: Context weight loss × Type: grocery vouchers	0.38*** [0.16, 0.60]
Model based on *n* observations (*n* participants)	8981 (450)

*P*-values based on Wald tests. *** < 0.001.

**Table 6:** Coefficients of models of choices about smoking cessation treatment and weight loss treatment including participants' smoking and overweight status respectively (Study 3).

Smoking cessation		Weight loss	
Factor/factor level	Coefficient [95% CI]	Factor/factor level	Coefficient [95% CI]
Intercept	−1.94*** [−2.30; −1.57]		−0.73*** [−1.09; −0.36]
Effectiveness: 5%	−1.83*** [−2.12; −1.54]		−2.10*** [-2.36; −1.84]
Effectiveness: 11%	0.62*** [0.37; 0.86]		0.64*** [0.40; 0.89]
Effectiveness: 20%	1.31*** [1.06; 1.56]		1.28*** [1.02; 1.54]
Effectiveness: 40%	1.87*** [1.62; 2.13]		1.80*** [1.52; 2.08]
Type: grocery vouchers	1.46*** [1.29; 1.63]		1.81*** [1.63; 1.99]
Smoking: have quit	−0.11 [−0.63; 0.41]	Overweight: formerly	0.59 [−0.13; 1.31]
Smoking: not daily	0.24 [−0.84; 1.33]	Overweight: slightly	0.21 [−0.26; 0.68]
Smoking: daily	0.69* [0.12; 1.26]	Overweight: very	0.93* [0.19; 1.66]
Model based on *n* observations (*n* participants)	4419 (443)		4402 (441)

*P*-values based on Wald tests. *** < 0.001; * < 0.05.
