# Supplementary material for: “Pay them if it works”: Discrete choice experiments on the acceptability of financial incentives to change health related behaviour
Source: Soc Sci Med. 2012 Dec;75(12):2509–14. doi: 10.1016/j.socscimed.2012.09.033 (PMC3686527; doi:10.1016/j.socscimed.2012.09.033)
Supplement: Supplementary file 1 [file mmc1.pdf]

Choice task introductions

Study 1:

“We will show you some treatments for smoking cessation. We will show you pairs of such treatments and ask you to pick one. For each pair, please pick the treatment you think should be funded if both were available but only one could be funded.”

Study 2:

“We will show you some treatments for weight loss. We will show you pairs of such treatments and ask you to pick one. For each pair, please pick the treatment you think should be funded if both were available but only one could be funded.”

Study 3:

1st choice block:

“You will see pairs of treatments for smokers to stop smoking [weight loss for overweight people].

For each pair, please pick the treatment you think should be funded.

The treatments

- differ in type
- differ in how effective they are.

You will be shown 11 pairs of treatments. These pairs are in no particular order.

Please read each pair on its own before choosing.”

2nd choice block:

“You will now see pairs of treatments for a different problem:

treatments for weight loss for overweight people [smokers to stop smoking].

The task is otherwise the same as what you've been doing so far.

For each pair, please pick the treatment you think should be funded.”
