# Supplementary material for: “Pay them if it works”: Discrete choice experiments on the acceptability of financial incentives to change health related behaviour
Source: Soc Sci Med. 2012 Dec;75(12):2509–14. doi: 10.1016/j.socscimed.2012.09.033 (PMC3686527; doi:10.1016/j.socscimed.2012.09.033)
Supplement: Supplementary file 2 [file mmc2.zip › S-2 Material Study 1.html]

#
