# Supplementary material for: “Pay them if it works”: Discrete choice experiments on the acceptability of financial incentives to change health related behaviour
Source: Soc Sci Med. 2012 Dec;75(12):2509–14. doi: 10.1016/j.socscimed.2012.09.033 (PMC3686527; doi:10.1016/j.socscimed.2012.09.033)
Supplement: Supplementary file 4 [file mmc4.pdf]

Healthcare treatments

What the public thinks

Public views on new treatments

You will see pairs of treatments for smokers to stop smoking.

For each pair, please pick the treatment you think should be funded.

The treatments

- differ in type
- differ in how effective they are

You will be shown 11 pairs of treatments.

These pairs are in no particular order.

Please read each pair on its own before choosing.

**Treatment A**	**Treatment B**
The patient receives	The patient receives
**standard medication**	**vouchers for healthy groceries**
worth £50 per month<br>for stopping smoking.	worth £50 per month<br>for not smoking.
This medication is proven to help	This intervention is proven to help
**10 out of 100 treated**.	**10 out of 100 treated**.

Which treatment should be funded?

- ☐ Treatment A
- ☐ Treatment B

**Treatment A**	**Treatment B**
The patient is paid	The patient receives
**cash**	**standard medication**
£50 per month<br>for not smoking.	worth £50 per month<br>for stopping smoking.
This intervention is proven to help	This medication is proven to help
**20 out of 100 treated**.	**10 out of 100 treated**.

Which treatment should be funded?

☐ Treatment A
☐ Treatment B

**Treatment A**	**Treatment B**
The patient receives<br>**standard medication**<br>worth £50 per month<br>for stopping smoking.<br><br>This medication is proven to help<br>**10 out of 100 treated**.	The patient is paid<br>**cash**<br>£50 per month<br>for not smoking.<br><br>This intervention is proven to help<br>**5 out of 100 treated**.

Which treatment should be funded?

- ☐ Treatment A
- ☐ Treatment B

**Treatment A**	**Treatment B**
The patient receives	The patient receives
**vouchers for healthy groceries**	**standard medication**
worth £50 per month for not smoking.	worth £50 per month for stopping smoking.
This intervention is proven to help	This medication is proven to help
**20 out of 100 treated**.	**10 out of 100 treated**.

Which treatment should be funded?

- ☐ Treatment A
- ☐ Treatment B

**Treatment A**	**Treatment B**
The patient receives<br>**standard medication**<br>worth £50 per month<br>for stopping smoking.<br><br>This medication is proven to help<br>**10 out of 100 treated**.	The patient is paid<br>**cash**<br>£50 per month<br>for not smoking.<br><br>This intervention is proven to help<br>**11 out of 100 treated**.

Which treatment should be funded?

☐ Treatment A
☐ Treatment B

Treatment A	Treatment B
The patient receives	The patient receives
**vouchers for healthy groceries**	**standard medication**
worth £50 per month for not smoking.	worth £50 per month for stopping smoking.
This intervention is proven to help	This medication is proven to help
**40 out of 100 treated**.	**10 out of 100 treated**.

Which treatment should be funded?

- ☐ Treatment A
- ☐ Treatment B

Treatment A	Treatment B
The patient receives **standard medication** worth £50 per month for stopping smoking. This medication is proven to help **10 out of 100 treated**.	The patient receives **standard medication** worth £50 per month for stopping smoking. This medication is proven to help **20 out of 100 treated**.

Which treatment should be funded?

☐ Treatment A
☐ Treatment B

**Treatment A**	**Treatment B**
The patient receives **vouchers for healthy groceries** worth £50 per month for not smoking. This intervention is proven to help **5 out of 100 treated**.	The patient receives **standard medication** worth £50 per month for stopping smoking. This medication is proven to help **10 out of 100 treated**.

Which treatment should be funded?

☐ Treatment A
☐ Treatment B

Treatment A	Treatment B
The patient receives	The patient is paid
**standard medication**	**cash**
worth £50 per month<br>for stopping smoking.	£50 per month<br>for not smoking.
This medication is proven to help	This intervention is proven to help
**10 out of 100 treated**.	**40 out of 100 treated**.

Which treatment should be funded?

- ☐ Treatment A
- ☐ Treatment B

**Treatment A**	**Treatment B**
The patient receives<br>**vouchers for healthy groceries**<br>worth £50 per month<br>for not smoking.<br><br>This intervention is proven to help<br>**11 out of 100 treated**.	The patient receives<br>**standard medication**<br>worth £50 per month<br>for stopping smoking.<br><br>This medication is proven to help<br>**10 out of 100 treated**.

Which treatment should be funded?

☐ Treatment A
☐ Treatment B

Treatment A	Treatment B
The patient receives	The patient is paid
**standard medication**	**cash**
worth £50 per month<br>for stopping smoking.	£50 per month<br>for not smoking.
This medication is proven to help	This intervention is proven to help
**10 out of 100 treated**.	**10 out of 100 treated**.

Which treatment should be funded?

☐ Treatment A
☐ Treatment B

You will now see pairs of treatments for a different problem:

treatments for weight loss for overweight people.

The task is otherwise the same as what you've been doing so far.

For each pair, please pick the treatment you think should be funded.

**Treatment A**	**Treatment B**
The patient receives	The patient is paid
**standard treatment**	**cash**
worth £50 per month for weight loss.	£50 per month for meeting weight loss targets.
This treatment is proven to help	This intervention is proven to help
**10 out of 100 treated**.	**20 out of 100 treated**.

Which treatment should be funded?

- ☐ Treatment A
- ☐ Treatment B

**Treatment A**	**Treatment B**
The patient is paid<br>**cash**<br>£50 per month<br>for meeting weight loss targets.<br><br>This intervention is proven to help<br>**11 out of 100 treated**.	The patient receives<br>**standard treatment**<br>worth £50 per month<br>for weight loss.<br><br>This treatment is proven to help<br>**10 out of 100 treated**.

Which treatment should be funded?

☐ Treatment A
☐ Treatment B

Treatment A	Treatment B
The patient receives	The patient is paid
**standard treatment**	**cash**
worth £50 per month<br>for weight loss.	£50 per month<br>for meeting weight loss targets.
This treatment is proven to help	This intervention is proven to help
**10 out of 100 treated**.	**10 out of 100 treated**.

Which treatment should be funded?

- ☐ Treatment A
- ☐ Treatment B

**Treatment A**	**Treatment B**
The patient receives<br>**vouchers for healthy groceries**<br>worth £50 per month<br>for meeting weight loss targets.<br><br>This intervention is proven to help<br>**20 out of 100 treated**.	The patient receives<br>**standard treatment**<br>worth £50 per month<br>for weight loss.<br><br>This treatment is proven to help<br>**10 out of 100 treated**.

Which treatment should be funded?

- ☐ Treatment A
- ☐ Treatment B

Treatment A	Treatment B
The patient receives<br>**standard treatment**<br>worth £50 per month<br>for weight loss.<br><br>This treatment is proven to help<br>**10 out of 100 treated**.	The patient receives<br>**vouchers for healthy groceries**<br>worth £50 per month<br>for meeting weight loss targets.<br><br>This intervention is proven to help<br>**11 out of 100 treated**.

Which treatment should be funded?

- ☐ Treatment A
- ☐ Treatment B

**Treatment A**	**Treatment B**
The patient receives<br>**standard treatment**<br>worth £50 per month<br>for weight loss.<br><br>This treatment is proven to help<br>**20 out of 100 treated**.	The patient receives<br>**standard treatment**<br>worth £50 per month<br>for weight loss.<br><br>This treatment is proven to help<br>**10 out of 100 treated**.

Which treatment should be funded?

- ☐ Treatment A
- ☐ Treatment B

**Treatment A**	**Treatment B**
The patient receives<br>**standard treatment**<br>worth £50 per month<br>for weight loss.<br><br>This treatment is proven to help<br>**10 out of 100 treated**.	The patient is paid<br>**cash**<br>£50 per month<br>for meeting weight loss targets.<br><br>This intervention is proven to help<br>**40 out of 100 treated**.

Which treatment should be funded?

- ☐ Treatment A
- ☐ Treatment B

**Treatment A**	**Treatment B**
The patient receives	The patient receives
**vouchers for healthy groceries**	**standard treatment**
worth £50 per month<br>for meeting weight loss targets.	worth £50 per month<br>for weight loss.
This intervention is proven to help	This treatment is proven to help
**10 out of 100 treated**.	**10 out of 100 treated**.

Which treatment should be funded?

- ☐ Treatment A
- ☐ Treatment B

**Treatment A**	**Treatment B**
The patient receives<br>**standard treatment**<br>worth £50 per month<br>for weight loss.<br><br>This treatment is proven to help<br>**10 out of 100 treated**.	The patient is paid<br>**cash**<br>£50 per month<br>for meeting weight loss targets.<br><br>This intervention is proven to help<br>**5 out of 100 treated**.

Which treatment should be funded?

- ☐ Treatment A
- ☐ Treatment B

**Treatment A**	**Treatment B**
The patient receives	The patient receives
**vouchers for healthy groceries**	**standard treatment**
worth £50 per month<br>for meeting weight loss targets.	worth £50 per month<br>for weight loss.
This intervention is proven to help	This treatment is proven to help
**40 out of 100 treated**.	**10 out of 100 treated**.

Which treatment should be funded?

☐ Treatment A
☐ Treatment B

**Treatment A**	**Treatment B**
The patient receives<br>**standard treatment**<br>worth £50 per month<br>for weight loss.<br><br>This treatment is proven to help<br>**10 out of 100 treated**.	The patient receives<br>**vouchers for healthy groceries**<br>worth £50 per month<br>for meeting weight loss targets.<br><br>This intervention is proven to help<br>**5 out of 100 treated**.

Which treatment should be funded?

☐ Treatment A
☐ Treatment B

What do you think about the NHS paying overweight people cash if they lose weight?

Which of the following four statements best describes your view?

- ☐ This is acceptable if it leads to benefits that outweigh any negative effects.
- ☐ This would be acceptable if it leads to benefits that outweigh any negative effects, but I don't think this is the case in the real world.
- ☐ I can imagine no benefits that could ever outweigh the negative effects this has, even in an ideal world, so it is not acceptable.
- ☐ This is not acceptable even if the benefits were to outweigh any negative effects.

What do you think about the NHS paying smokers cash to stop smoking?

Which of the following four statements best describes your view?

- ☐ This is acceptable if it leads to benefits that outweigh any negative effects.
- ☐ This would be acceptable if it leads to benefits that outweigh any negative effects, but I don't think this is the case in the real world.
- ☐ I can imagine no benefits that could ever outweigh the negative effects this has, even in an ideal world, so it is not acceptable.
- ☐ This is not acceptable even if the benefits were to outweigh any negative effects.

How responsible do you think smokers are for smoking?

Not at all responsible □—□—□—□—□—□—□ Entirely responsible

How responsible do you think overweight people are for being overweight?

Not at all responsible □—□—□—□—□—□—□ Entirely responsible

People in our society often disagree about how far to let individuals go in making decisions for themselves. How strongly do you agree or disagree with each of these statements?

	strongly disagree	moderately disagree	slightly disagree	slightly agree	moderately agree	strongly agree
The government interferes far too much in our everyday lives.	☐	☐	☐	☐	☐	☐
Sometimes government needs to make laws that keep people from hurting themselves.	☐	☐	☐	☐	☐	☐
It's not the government's business to try to protect people from themselves.	☐	☐	☐	☐	☐	☐
The government should stop telling people how to live their lives.	☐	☐	☐	☐	☐	☐
The government should do more to advance society's goals, even if that means limiting the freedom and choices of individuals.	☐	☐	☐	☐	☐	☐
Government should put limits on the choices individuals can make so they don't get in the way of what's good for society.	☐	☐	☐	☐	☐	☐

People in our society often disagree about issues of equality and discrimination. How strongly do you agree or disagree with each of these statements?

	strongly disagree	moderately disagree	slightly disagree	slightly agree	moderately agree	strongly agree
We have gone too far in pushing equal rights in this country.	☐	☐	☐	☐	☐	☐
Our society would be better off if the distribution of wealth was more equal.	☐	☐	☐	☐	☐	☐
We need to dramatically reduce inequalities between the rich and the poor, whites and people of color, and men and women.	☐	☐	☐	☐	☐	☐
Discrimination against minorities is still a very serious problem in our society.	☐	☐	☐	☐	☐	☐
It seems like blacks, women, homosexuals and other groups don't want equal rights, they want special rights just for them.	☐	☐	☐	☐	☐	☐
Society as a whole has become too soft and feminine.	☐	☐	☐	☐	☐	☐

Imagine the NHS would offer you a choice of interventions to help you improve your health through behaviour change. Which type of intervention would **you personally choose**?

Please rank the interventions from 1 to 5, where **1 is your most preferred** and **5 is your least preferred** option.

Please use each number (1–5) once, to assign a rank to each option:

______ Medication

______ Standard behavioural treatment

______ An incentive scheme paying vouchers for luxury items

______ An incentive scheme paying vouchers for healthy groceries

______ An incentive scheme paying cash

Now think about **other people** to whom the NHS would offer the same type of interventions. Which type of intervention do you think **they** would choose?

1 is what you think is **most preferred by others** and **5** is what you think is **least preferred** by others:

______ Medication

______ Standard behavioural treatment

______ An incentive scheme paying vouchers for luxury items

______ An incentive scheme paying vouchers for healthy groceries

______ An incentive scheme paying cash

We would welcome your comments about why you chose between the different treatments the way you did.

(Please indicate whether what you write applies to smoking cessation, or weight loss, or both).

Please tell us about yourself

This research is more meaningful if we can tell whether those who took part reflect the population reasonably well.

What is your gender? ☐ Male ☐ Female

How old are you? ____________ years old

What is your highest level of educational qualification?

☐ No qualifications
☐ GCSE or similar
☐ GCE A level or similar
☐ Higher education or similar
☐ Degree or similar
☐ Other

Are you or have you ever been overweight?

☐ I am currently very overweight.
☐ I am currently slightly overweight.
☐ I used to be overweight but am now normal weight.
☐ I have never been overweight.
☐ Don't know or prefer not to answer.

Do you smoke or have you smoked?

☐ I currently smoke every day.
☐ I currently smoke, but not every day.
☐ I used to smoke but have quit.
☐ I have never smoked.
☐ I prefer not to answer.

What is your postcode? ____________

Thank you!

v1
smo g10-c20-c5-g20-c11-g40-m20-g5-c40-g11-c10
wei c20-c11-c10-g20-g11-m20-c40-g10-c5-g40-g5
