# Supplementary material for: “Pay them if it works”: Discrete choice experiments on the acceptability of financial incentives to change health related behaviour
Source: Soc Sci Med. 2012 Dec;75(12):2509–14. doi: 10.1016/j.socscimed.2012.09.033 (PMC3686527; doi:10.1016/j.socscimed.2012.09.033)
Supplement: Supplementary file 5 [file mmc5.pdf]

Internal consistency

We had two tests of participants' internal consistency. They help us identify participants who failed to understand the task or failed to take it seriously and need to be excluded from the study.

Dominance check. We had included one choice pair with a dominant option (for Study 3, once per treatment context); participants were expected to choose standard treatment at 20% effectiveness over standard treatment at 10% effectiveness.

Intransitive choices. A participant's choice is intransitive if he chooses an incentive type at a lower level of effectiveness but rejects it at a higher level of effectiveness. Not necessarily a sign of lack of interest or understanding, this can also happen because of true indifference between options. We follow Ryan and Farrar's (2000) recommendation that inconsistencies merit closer inspection and where necessary look at it in conjunction with other information; see below.

Study 1

We excluded three participants who had two intransitive choices; one of them also failed a dominance check and had a very fast study completion time. Nobody else failed the dominance check.

Study 2

We excluded two participants who had very fast study completion time; they had one and two intransitive choices respectively. Nobody else failed the dominance check or had more than one intransitive choice.

Study 3

Dominance check. 419 participants (81%) passed this test in both treatment contexts; 79 participants (15%) pass it once; 22 (4%) fail it both times. Omitting the question was counted as

passing; this affected nine participants. We excluded all participants who did not pass the test both times. We look more closely at those who fail once, below.

Intransitive choices. We combine information about intransitive choices and use the dominance check to identify low quality data: we exclude only those 48 participants who have at least one intransitive choice and also fail a dominance check.

This means we keep participants who fail one dominance check but have no intransitive choices, and participants who have intransitive choices but pass both dominance checks, thus granting participants a small margin of error across the 22 choice pairs. We also estimated the model excluding all who failed at least one dominance check and obtain qualitatively identical results.

The remaining 450 participants were between 17 and 84 years old (SD = 17·53); 48% were women, very similar to our original sample.
